# Increased SHP-1 expression results in radioresistance, inhibition of cellular senescence, and cell cycle redistribution in nasopharyngeal carcinoma cells

**DOI:** 10.1186/s13014-015-0445-1

**Published:** 2015-07-28

**Authors:** Ziyi Sun, Xiaofen Pan, Zhenwei Zou, Qian Ding, Gang Wu, Gang Peng

**Affiliations:** Cancer Center, Union hosipital, Wuhan, 430022 Hubei Province China; Cancer center, Affliated Hospital of Guangdong Medical College, Zhanjiang, 524001 Guangdong Province China

**Keywords:** Nasopharyngeal carcinoma, SHP-1, Cellular senescence, Cell cycle distribution, p16, Rb

## Abstract

**Background:**

Radioresistance is the main limit to the efficacy of radiotherapy in nasopharyngeal carcinoma (NPC). SHP-1 is involved in cancer progression, but its role in radioresistance and senescence of NPC is not well understood. This study aimed to assess the role of SHP-1 in the radioresistance and senescence of NPC cells.

**Methods:**

SHP-1 was knocked-down and overexpressed in CNE-1 and CNE-2 cells using lentiviruses. Cells were irradiated to observe their radiosensitivity by colony forming assay. BrdU incorporation assay and flow cytometry were used to monitor cell cycle. A β-galactosidase assay was used to assess senescence. Western blot was used to assess SHP-1, p21, p53, pRb, Rb, H3K9Me3, HP1γ, CDK4, cyclin D1, cyclin E, and p16 protein expressions.

**Results:**

Compared with CNE-1-scramble shRNA cells, SHP-1 downregulation resulted in increased senescence (+107 %, *P* < 0.001), increased radiosensitivity, higher proportion of cells in G0/G1 (+33 %, *P* < 0.001), decreased expressions of CDK4 (−44 %, *P* < 0.001), cyclin D1 (−41 %, *P* = 0.001), cyclin E (−97 %, *P* < 0.001), Rb (−79 %, *P* < 0.001), and pRb (−76 %, *P* = 0.001), and increased expression of p16 (+120 %, *P* = 0.02). Furthermore, SHP-1 overexpression resulted in radioresistance, inhibition of cellular senescence, and cell cycle arrest in the S phase. Levels of p53 and p21 were unchanged in both cell lines (all *P* > 0.05).

**Conclusion:**

SHP-1 has a critical role in radioresistance, cell cycle progression, and senescence of NPC cells. Down-regulating SHP-1 may be a promising therapeutic approach for treating patients with NPC.

## Background

Nasopharyngeal carcinoma (NPC) is a cancer with a distinctly skewed geographic and ethnic distribution, and is endemic in Southern China and South East Asia [1]. Indeed, type I NPC represents 25 % of the cases in North America and 2 % in Southern China, while type III represents 95 % of the cases in China and 63 % in North America [1]. NPC mostly affects men (men:women ratio of 4.4:1), and the median age at diagnosis is in the early 60s. Worldwide incidence is <1 per 100,000 for either men or women, but the incidence is 20–30 per 100,000 in Hong Kong and 15–20 per 100,000 in Guangdong [1]. Tobacco and alcohol are the two main risk factors for NPC [2–4]. Human papillomavirus and Epstein-Barr virus also increase the risk of NPC, particularly in endemic regions [5, 6]. Mean survival of patients with stage I, II, or III NPC is about 3 years [7].

Radiotherapy (RT) is the main treatment for NPC since radical resection is typically not possible [4]. The advent of megavoltage radiotherapy has transformed a once lethal cancer into one that is readily curable. Non-keratinizing undifferentiated carcinoma (type III NPC) is the most common of the three forms of NPC, and is sensitive to radiations [4]. Overall survival exceeding 50 % at five years may now be achieved [8, 9].

However, radioresistance is the main factor limiting the benefits from radiotherapy [10]. The mechanisms of radioresistance are mostly unknown. Recent studies using microarrays have explored the genes involved in the radioresistance of solid tumors such as cervix, pancreas, mouth, lung, and esophagus and failed to identify common genes [11]. Nevertheless, recent studies have shown that a number of proteins involved in the cell cycle (gp96, GDF15, PTEN) were involved in the radioresistance of NPC [11, 12].

SHP-1 (initially designated as SHPTP-1, SHP, HCP, and PTPIC) is a cytosolic protein tyrosine phosphatase expressed primarily in hematopoietic cells [13]. SHP-1 has been proposed as a candidate tumor suppressor gene in lymphoma and solid cancers [14]. SHP-1 can play either negative or positive roles in regulating signal transduction pathways and is differentially expressed in a number of cancer cell lines [14–16]. Therefore, SHP-1 appears to have different roles and mechanisms in the regulation of cell cycle and cell proliferation in different types of tumors.

Recent data validated the early idea that cellular senescence is important for tumor suppression [17, 18]. Cellular senescence is a barrier to tumorigenesis and contributes to mammalian aging [19]. Furthermore, cellular senescence depends critically on two powerful tumor suppressor pathways: the p53 and pRb/p16INK4a pathways. These pathways are known to regulate cellular senescence/immortalization including the p16INK4a/pRB, p19ARF/p53/p21CIP1/WAF1, and PTEN/p27KIP1 pathways [20–22].

A previous study has shown that SHP-1 knockdown resulted in a G1/S arrest and an increase in the expression of p16 [23]. This previous study investigated the association between SHP-1 and p16, since p16 has previously been demonstrated to be silenced in the vast majority of NPCs [24], which suggested that SHP-1 may regulate cellular senescence through the p16 pathway.

Therefore, the aim of the present study was to study the association between expression of SHP-1 and cellular senescence, radioresistance and cell cycle distribution in NPC cells. This study is the first to propose that SHP-1 regulates a senescence response.

## Methods

### Cell culture

The human NPC cell lines CNE-1 and CNE-2 were obtained from the Cell Bank of Sun Yat-sen University (Guangzhou, China), and cultured in RPMI 1640 (Invitrogen Inc., Carlsbad, CA, USA) supplemented with 10 % fetal bovine serum (FBS, Invitrogen Inc., Carlsbad, CA, USA) and 1 % penicillin/streptomycin (Invitrogen Inc., Carlsbad, CA, USA). Cells were kept at 37 °C in 5 % CO_2_ atmosphere. The CNE-1 cell line was established in 1978 from a patient with NPC [25], and the CNE-2 cell line was established in 1983 from a poorly differentiated NPC [26]. The CNE-2 cell line has been shown to be less radio-resistant than CNE-1 [27], and the DNA repair mechanisms seem to be more efficient in the CNE-1 cell line [27, 28].

### SHP-1 overexpression and knockdown mediated by lentiviruses

For SHP-1 knockdown, cells were plated in 24-well plates and cultured with 0.5 ml of RPMI 1640 supplemented with 5 % FBS and 1 % penicillin/streptomycin for 24 h. Cells were then transduced with 50 μl (8.6 × 10^9^ copies/ml) of lentivirus-mediated SHP-1-shRNA vector (lot: LP-HSH015860-LVRH1MP) and scramble shRNA vector (lot: LP-CSHCTR001-LVRH1MP) (GeneCopoeia, Guangzhou, China) for 48 h. These vectors contained a puromycin resistance gene for the selection of transduced cells. Then, the transduced cells were digested with trypsin, plated in 6-well plates, and cultured in RPMI 1640 supplemented with 10 % FBS, 1 % penicillin/streptomycin and 2 μg/ml of puromycin for 12 days to screen for stably transduced cells. The medium containing puromycin was changed every three days. Puromycin-resistant clones were selected. SHP-1 mRNA and protein expressions were determined by real-time RT-PCR and western blot.

For SHP-1 overexpression, cells were plated in 24-well plates and cultured with 0.5 ml of RPMI 1640 supplemented with 15 % FBS and 1 % penicillin/streptomycin for 24 h. Cells were then transduced with 50 μl (8.6 × 10^9^ copies/ml) of lentivirus-mediated SHP-1-overexpression vector (lot: LP-H1802-Lv201-C0010) and scramble shRNA vector (lot: LP-NEG-Lv201-0200) (GeneCopoeia, Guangzhou, China) for 48 h. These vectors contained a puromycin resistance gene for the selection of transduced cells. Then, the transduced cells were observed using a Zeiss Axioplan 2 fluorescence microscope (Carl Zeiss GmbH, Oberkochen, Germany) equipped with a Plan Neofluar 20x/0.5 objective, color camera Infinity X and Deltapix software (DeltaPix, Nibe, Denmark). Transduced cells were digested with trypsin, plated in 6-well plates, and cultured in RPMI 1640 supplemented with 15 % FBS, 1 % penicillin streptomycin and 2 μg/ml of puromycin for 12 days to screen for stably transduced cells. The medium containing puromycin was changed every three days. Puromycin-resistant clones were selected. SHP-1 mRNA and protein expressions were determined by real-time RT-PCR and western blot.

### Colony forming assay

Cells were seeded in 6-well culture plates at different cell densities (200, 300, 600, 1500, and 4000 cells/well) and irradiated the next day using different doses (0, 2, 4, 6, and 8 Gy). The plates were incubated for 14 days, fixed with methanol, and stained with Giemsa (Sigma, St Louis, MI, USA). Colonies containing at least 50 cells were counted as a clone. A multi-target single-hit model was used to describe the survival fraction using the equation SF = 1-(1-e^-D/D0^)^N^, where SF is the cell survival fraction, D is the radiation dose, e is the natural logarithm, D0 is the mean lethal dose, and N is the extrapolated number. Survival curves were made using these SF.

### BrdU incorporation assay

Cells were cultured on glass slides and were incubated with 10 μM BrdU (Sigma, St Louis, MI, USA) for 6 h before fixation with 4 % formaldehyde. After DNA denaturation in 2 M HCl for 30 min, cells were washed in PBS and incubated with mouse anti-human primary antibody against BrdU (RPN20AB, 1:300, AP-Biotech S.R.L., Buenos Aires, Argentica). The secondary antibody Alexa Fluor® 568 goat anti-mouse (#A110-31, 1:500, Invitrogen Inc., Carlsbad, CA, USA) was then applied for 60 min at room temperature, followed by a final wash in PBS. Glass slides were mounted in Vectashield Mounting Medium with HOCHEST (Vector Laboratories, Burlingame, CA, USA). Images were acquired using an AxioObserver fluorescence microscope (Carl Zeiss GmbH, Oberkochen, Germany) equipped with a Plan Apochromat 20x/0.8 objective, camera Coolsnap HQ (Photometrics, Tucson, AZ, USA) and the Metamorph software (Universal Imaging, Bedford Hills, NY, USA). Two independent observers blinded to grouping counted the BrdU-positive cells in 35 random fields (approximately 500 cells) for each glass slide, and the percentage of BrdU-positive cells was calculated. Data analysis was performed using ImageJ 1.43u (National Institutes of Health, Bethesda, MD, USA).

### Senescence-associated β-galactosidase

Staining for senescence-associated-β-galactosidase activity was performed using a Senescence β-Galactosidase Staining Kit (#9860, Cell Signaling, Danvers, MA, USA) according to the manufacturer’s protocol. β-galactosidase positive cells (green) were viewed by light microscopy.

### Immunofluorescence

For immunofluorescence, cells were cultured on glass slides, fixed in 4 % formaldehyde and permeabilized by 0.1 % Triton X-100 in two consecutive steps, each for 15 min at room temperature. After washing with PBS, cells were blocked for 30 min in 10 % fetal calf serum. The following primary antibodies were used: rabbit anti-human polyclonal antibody against histone H3 trimethylated at lysine 9 (H3K9Me3, #07-442, 1:1,000, Millipore corp., Billerica, MA, USA) and mouse anti-human polyclonal antibody against heterochromatin protein-1γ (HP1γ, #MAB3450, 1:4,000, Millipore corp., Billerica, MA, USA). Incubation with the primary antibodies was performed for 60 min at room temperature and the cells were washed with PBS. The secondary antibodies Alexa Fluor® 568 goat anti-mouse (#A110-31, 1:500, Invitrogen Inc., Carlsbad, CA, USA) and Alexa Fluor® 488 goat anti-rabbit (#A-11008, 1:500, Invitrogen Inc., Carlsbad, CA, USA) were then applied for 60 min at room temperature, followed by a final wash in PBS. Glass slides were mounted in Vectashield Mounting Medium with HOCHEST (Vector Laboratories, Burlingame, CA, USA). Confocal images were acquired using a LSM-510 microscope (Carl Zeiss GmbH, Oberkochen, Germany) equipped with a Plan-Apochromat 63x/1.4 oil immersion objective and the ZEN2009 software (Carl Zeiss GmbH, Oberkochen, Germany). Identical image acquisition parameters were used for quantitative and comparative imaging. Percentage of senescence-associated heterochromatin foci (SAHF)-positive cells (based on Hoechst staining) and numbers of H3K9Me3 and HP1γ foci per cell were counted by two independent observers blinded to grouping from at least 200 cells from each glass slide. Data analysis was performed using ImageJ 1.43u (National Institutes of Health, Bethesda, MD, USA).

### Flow cytometry

After transduction with the indicated lentiviruses, cells were fixed overnight with 70 % ethanol, followed by resuspension in PBS containing 1 mg/ml of RNase and 50 μg/ml of propidium iodide (Sigma, St Louis, MI, USA). Cellular DNA content was determined using a FACScan flow cytometer (BD Biosciences, Franklin Lake, NJ, USA).

### Real-time RT-PCR

Total RNA was extracted using TRIzol (Invitrogen, NM, USA), and cDNA synthesis was performed using the Prime Script RT-PCR kit (Takara, Shiga, Japan), according to the manufacturers’ instructions. Then, real-time PCR was performed using SYBR Green PCR Master Mix (Applied Biosystems, USA) in a PCR amplifier (ABI Prism 7000, USA). The StepOne^TM^ Software v2.1 was used to analyze the data. The primer sequences for SHP-1 were: Forward, 5’-ACCATCATCCACCTCAAGTACC-3’ and Reverse, 5’-CTGAGCACAGAAAGCACGAA-3’. β-actin was used as an internal control, and the primer sequences were: Forward, 5’-GATGAGATTGGCATGGCTTT-3’ and Reverse, 5’-CACCTTCACCGTTCCAGTTT-3’. 2^-ΔΔCt^ was calculated to represent the relative mRNA expression of target genes.

### Western blot

Cells were lysed with the RIPA buffer (Invitrogen Inc., Carlsbad, CA, USA), and then centrifuged (12,000 rpm, 15 min, 4 °C). Protein content was measured using a BCA assay. Equal amounts of proteins (20–80 μg) were separated with 10 % sodium docecyl sulfate-polyacrylamide gel electrophoresis, and transferred to polyvinylidene difluoride membranes (Santa Cruz Biotechnology, Santa Cruz, CA, USA). Membranes were blocked with 5 % BSA for 1 h, and probed using mouse anti-human p21 (1:400; Abcam, Cambridge, MA, USA), mouse anti-human p53 (1:1000, Cell Signaling, Danvers, MA, USA), mouse anti-human pRb (Ser795) (1:1000, Cell Signaling, Danvers, MA, USA), mouse anti-human Rb (1:2000, Cell Signaling, Danvers, MA, USA), mouse anti-human H3K9Me3 (1:500, Millipore corp., Billerica, MA, USA), mouse anti-humanHP1-γ (1:500, Millipore corp., Billerica, MA, USA), mouse anti-human SHP1 (1:1000, Epitomic, San Francisco, CA, USA), mouse anti-human CDK4 (1:1000, Abcam, Cambridge, MA, USA), mouse anti-human cyclin D1 (1:1000, Epitomic, San Francisco, CA, USA), mouse anti-human cyclin E (1:1000, Bioworld Technology Inc., Louis Park, MN, USA), mouse anti-human p16 (1:1000, Santa Cruz Biotechnology, Santa Cruz, CA, USA), or rabbit anti-human β-actin (1:2500; Sigma, St Louis, MI, USA) polyclonal antibodies overnight at 4 °C. Primary antibodies were detected using horseradish peroxidase-conjugated secondary antibodies (Invitrogen Inc., Carlsbad, CA, USA), and were visualized using enhanced chemiluminescence (SuperSignal, Pierce, Rockford, IL, USA). Grayscale images were analyzed using ImageJ 1.43b (National Institutes of Health, Bethesda, MD, USA).

### Statistical analysis

Statistical analysis was performed using SPSS 12.0 (SPSS Inc., Chicago, IL, USA). Data are expressed as means ± standard deviation (SD) of at least three independent experiments and evaluated by one-way analysis of variance (ANOVA) with the least significant difference (LSD) test for post hoc analysis or student’s *t*-test. *P-*values <0.05 were considered statistically significant.

## Results

### Expression of SHP-1 by lentivirus-mediated RNA interference and overexpression

**Fig. 1:**
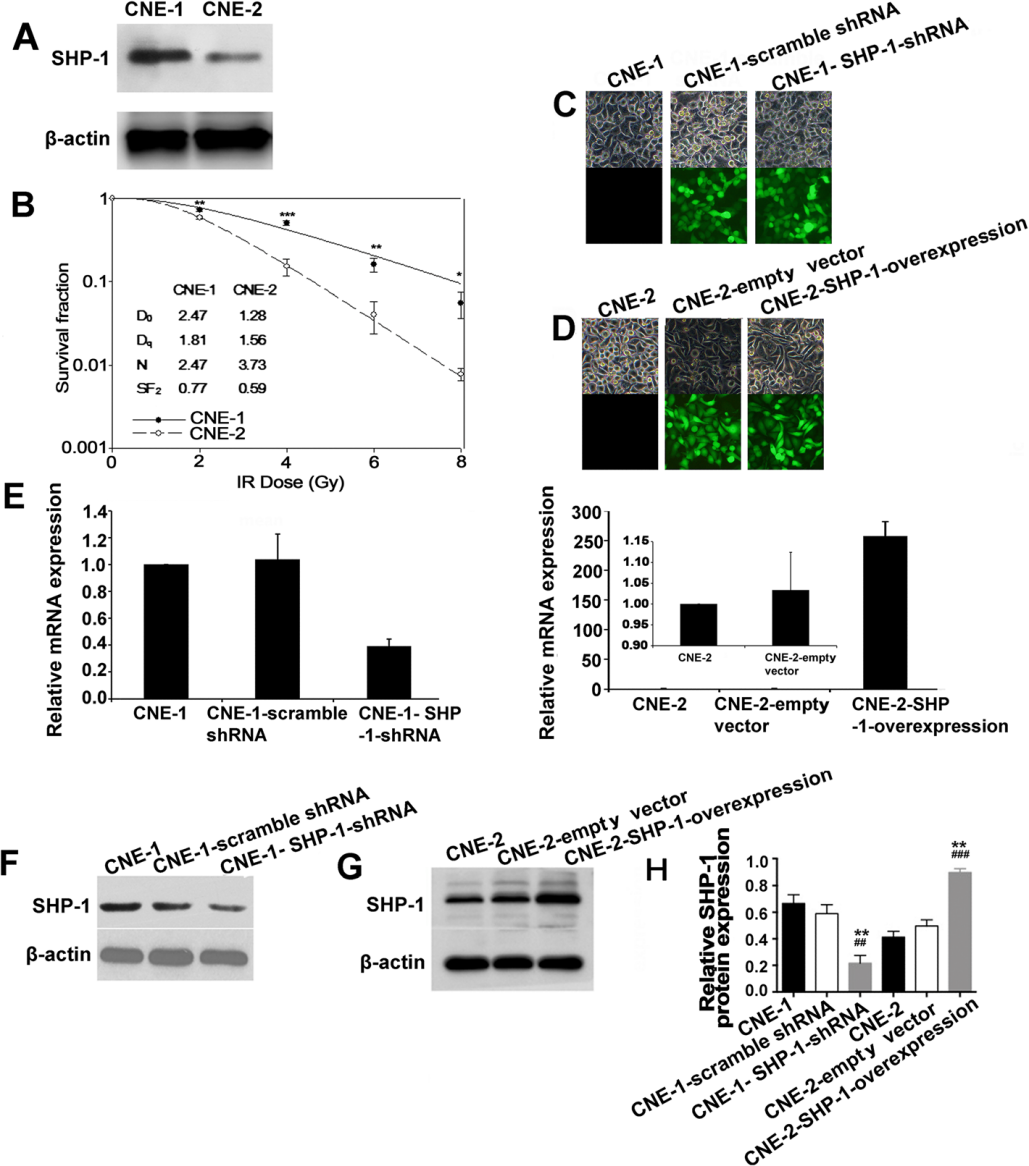
Alteration of SHP-1 expression in human nasopharyngeal carcinoma (NPC) cell lines CNE-1 and CNE-2 by lentivirus-mediated RNA interference and overexpression, respectively. **a** SHP-1 protein expression in CNE-1 and CNE-2 cells was determined by western blot. **b** CNE-1 and CNE-2 cell survival according to radiation dose determined by colony formation assay. *P < 0.05, **P < 0.01, ***P < 0.001 CNE-1 vs. CNE-2. CNE-1: non-transduced CNE-1 cells; CNE-1-scramble shRNA: CNE-1 cells transduced with lentivirus-mediated scramble shRNA; CNE-1 SHP-1 shRNA: CNE-1 cells transduced with lentivirus-mediated SHP-1 shRNA; CNE-2: without transduced CNE-2 cells; CNE-2-empty vector: CNE-2 cells transduced with lentivirus vector; CNE-2 SHP-1 overexpression: CNE-2 cells transduced with lentivirus-mediated SHP-1 overexpression. According to fluorescence microscopy, transduction efficiency in CNE-1 (**c**) and CNE-2 (**d**) cells was >90 % at 2 days after transduction (magnification: ×400). **e** SHP-1 mRNA expression were determined by real-time RT-PCR. Relative mRNA expression was normalized to CNE-1 or CNE-2, and β-actin was used as an inner control. SHP-1 protein expression in CNE-1 (**f**) and CNE-2 (**g**) cells was determined by western blot. β-actin was used as control. **h** Quantitative results of western blot are shown as mean ± standard deviation (SD) from three independent experiments. ^**^ *P* < 0.01, ^***^ *P* < 0.001 vs. CNE-1 or CNE-2; ^##^ *P* < 0.01, ^###^ *P* < 0.001 vs. CNE-1-scramble shRNA or CNE-2-empty vector

Figure 1a and b show the baseline expression of SHP-1 and radiosensitivity in CNE-1 and CNE-2 cells. The majority of cells above 90 % displayed green fluorescence 48 h after lentivirus transduction (Fig. 1c and d). Real-time RT-PCR showed that SHP-1 mRNA expression was suppressed by 62.5 % in CNE-1 cells transduced with lentivirus-mediated SHP-1 shRNA (CNE-1 SHP-1 shRNA), compared with cells transduced with lentivirus-mediated scramble shRNA (CNE-1-scramble shRNA), while SHP-1 mRNA expression was overexpressed 249.2 folds in CNE-2 cells transduced with lentivirus-mediated SHP-1 overexpression (CNE-2 SHP-1 overexpression), compared with cells transduced with lentivirus vector (CNE-2-empty vector) (Fig. 1e). Real-time RT-PCR results were confirmed by western blotting (Fig. 1f, g, and h), *i.e.* -47.2 % in CNE-1 SHP-1 shRNA cells, and +90.3 % in CNE-2 SHP-1 overexpression cells.

### Effects of SHP-1 knockdown and overexpression in CNE-1 and CNE-2 cells on radiosensitivity

**Fig. 2:**
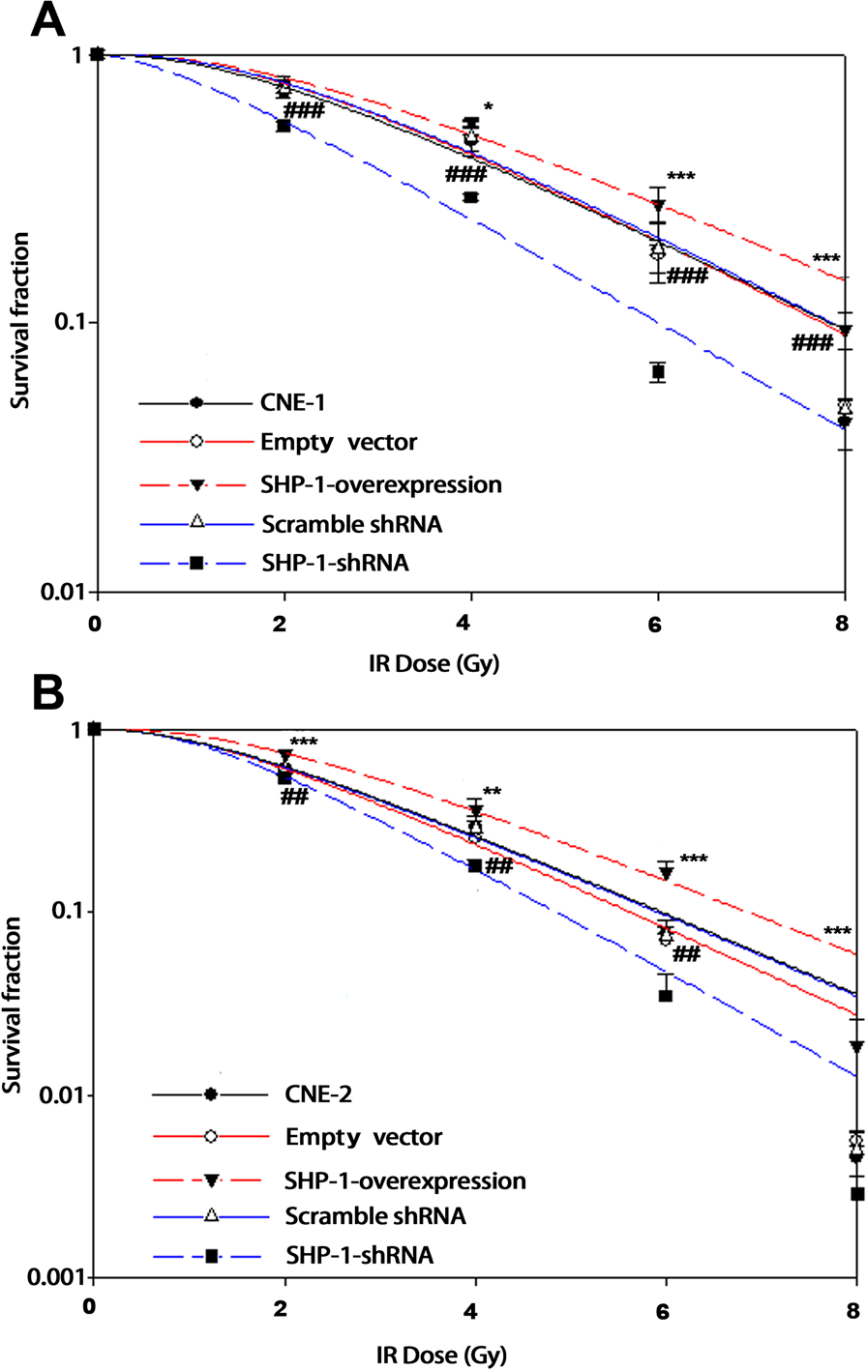
Effects of SHP-1 knockdown and overexpression on radiosensitivity in CNE-1 and CNE-2 cells. After stable transduction with lentivirus-mediated RNA interference and overexpression, CNE-1 (**a**) and CNE-2 (**b**) cells were irradiated at different doses (0, 2, 4, 6 and 8 Gy) (radiation absorption rate was 2 Gy/min). After further incubation for 14 days, colony formation assays were used to examine the radiosensitivity of the NPC cells. Survival curves were fitted according to the multi-target single-hit model. Compared with CNE-1 cells, cell survival was significantly different (all *P* < 0.05, ANOVA) for all radiation doses in CNE-1 cells with SHP-1 overexpression or silencing. Compared with CNE-2 cells, cell survival was significantly different (all *P* < 0.05, ANOVA) for all radiation doses in CNE-2 cells with SHP-1 overexpression or silencing. *P < 0.05, **P < 0.01, ***P < 0.001 empty vector vs. SHP-1-overexpression; ##*P* < 0.01, ###*P* < 0.001 scramble shRNA vs. SHP-1-shRNA

Survival curves of CNE-1, CNE-1-empty vector, CNE-1-scramble shRNA, CNE-1 SHP-1 shRNA, and CNE-1 SHP-1 overexpression cells after irradiation are shown in Fig. 2a, and the survival curves of CNE-2, CNE-2-empty vector, CNE-2-scramble shRNA, CNE-2 SHP-1 shRNA, and CNE-2 SHP-1 overexpression cells after irradiation are shown in Fig. 2b. The curves show that SHP-1 overexpression cells had a higher radioresistance compared with nontransduced cells or cells transduced with empty vector (*P* < 0.001 for all radiation doses) (Fig. 2). Notably, SHP-1 overexpression cells had higher D0, Dq, and SF2 values compared with non-transduced cells, indicating higher radioresistance (Table 1). By contrast, D0, Dq, and SF2 values were similar between the non-transduced cells and cells transduced with empty vector. On the other hand, SHP-1 shRNA cells had a lower radioresistance compared with non-transduced cells or cells transduced with scramble shRNA (*P* < 0.001 for all radiation doses) (Fig. 2 and Table 1).

**Table 1:** Effects of SHP-1 knockdown and overexpression in CNE-1 and CNE-2 cells on radiosensitivity parameters

Radiosensitivity parameters	CNE-1	CNE-1-empty vector	CNE-1- scramble shRNA	CNE-1- SHP-1-overexpression	CNE-1- SHP-1- shRNA	CNE-2	CNE-2- empty vector	CNE-2- scramble shRNA	CNE-2- SHP-1- overexpression	CNE-2- SHP-1- shRNA
SF2	0.75	0.78	0.78	0.82	0.56	0.62	0.60	0.61	0.74	0.55
D_0_	2.51	2.38	2.38	2.85	2.17	1.94	1.80	1.94	2.08	1.50
D_q_	1.77	1.85	1.85	1.96	1.26	1.46	1.43	1.43	1.75	1.36
N	2.34	2.68	2.65	2.48	1.62	2.22	2.32	2.17	2.77	2.58

***Note*** **:** SF2: cell survival fraction with 2 cGy irradiation dose; D0: mean lethal dose value; Dq: quasithreshold dose; N: extrapolation number

### Effects of SHP-1 knockdown in CNE-1 cells and overexpression in CNE-2 cells on NPC cell senescence

**Fig. 3:**
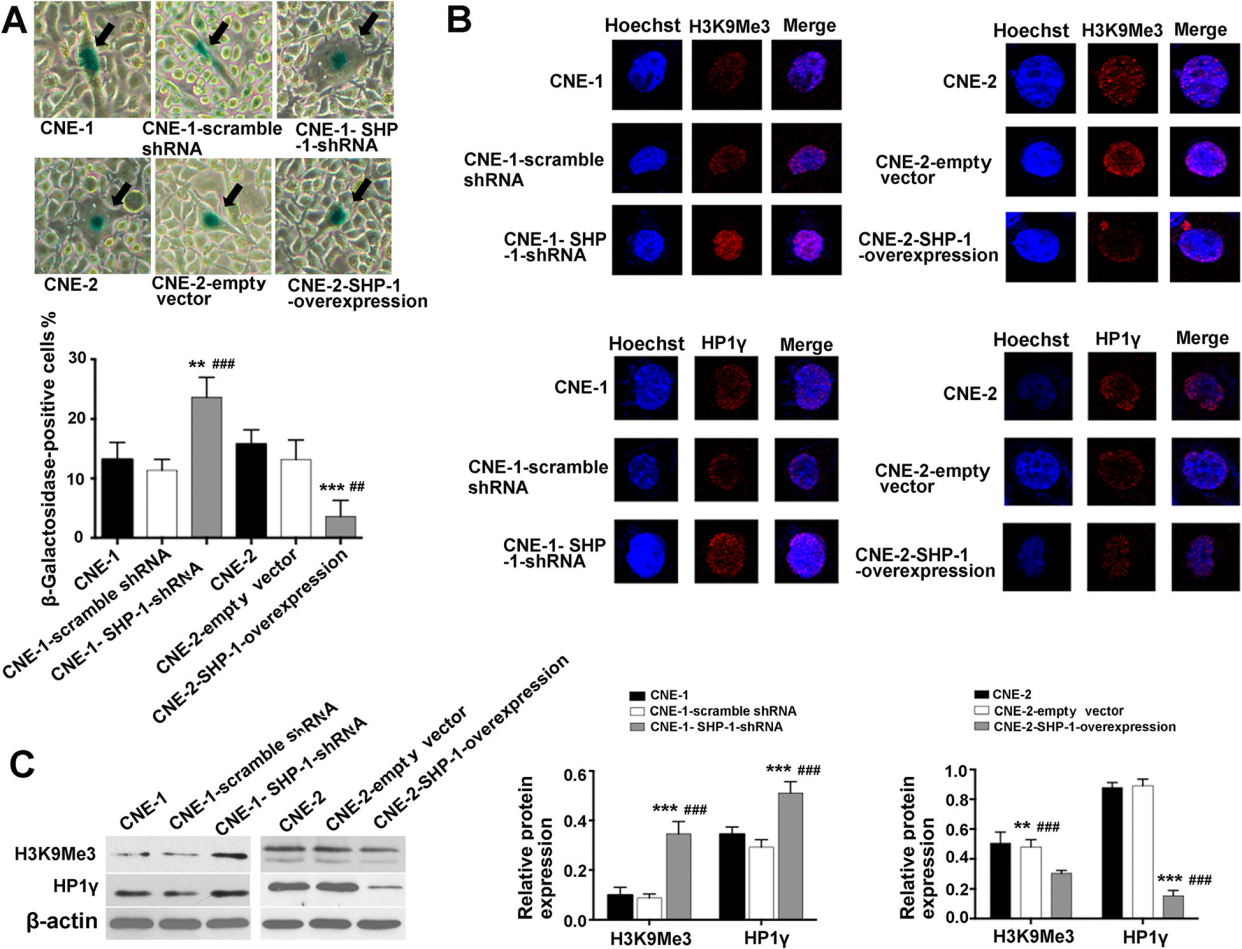
Effects of SHP-1 knockdown in CNE-1 cells and overexpression in CNE-2 cells on cell senescence. **a** Cell senescence was determined by β-galactosidase staining (magnification: ×400). Arrows: senescent cells. Heterochromatin markers H3K9Me3 and HP1γ location and protein expression were determined by immunofluorescence staining (**b**) (magnification: ×400; red: Alexa Fluor® 568) and western blot (**c**), respectively. β-actin was used as an inner control. Data are shown as mean ± SD. ^**^ *P* < 0.01, ^***^ *P* < 0.001 vs. CNE-1 or CNE-2; ^##^ *P* < 0.01, ^###^ *P* < 0.001 vs. CNE-1- scramble shRNA or CNE-2-empty vector

Cell cycle arrest is the central feature of senescent cells. Morphologically, CNE-2 and CNE-2-empty vector cells were vacuolated, flattened and much larger in size compared with CNE-2 SHP-1 overexpression cells (Fig. 3a). β-galactosidase staining revealed higher senescence in CNE-1 SHP-1 shRNA cells compared with CNE-1-scramble shRNA cells (23.6 ± 3.4 % vs. 11.4 ± 1.8 %, *P* < 0.001), and lower senescence in CNE-2 SHP-1 overexpression cells compared with CNE-2-empty vector cells (3.6 ± 2.7 % vs. 13.2 ± 3.3 %, *P =* 0.001) (Fig. 3a).

H3K9Me3 and HP1γ analyses showed that more CNE-1 SHP-1 shRNA cells were positive for H3K9Me3 (13.6 ± 2.7 % vs. 4.6 ± 1.9 %, *P* < 0.001) and HP1γ (12.0 ± 2.2 % vs. 2.8 ± 1.5 %, *P* < 0.001) compared with CNE-1-scramble shRNA cells, while fewer CNE-2 SHP-1 overexpression cells were positive for H3K9Me3 (2.6 ± 1.5 % vs. 9.6 ± 2.1 %, *P* < 0.001) and HP1γ (3.6 ± 1.5 % vs. 10.0 ± 2.3 %, *P* = 0.001) (Fig. 3b). These results were confirmed by western blot for H3K9Me3 and HP1γ, *i.e.* +292 % for H3K9Me3 and +54 % for HP1γ in CNE-1 SHP-1 shRNA cells compared with CNE-1-scramble shRNA cells, and −37 % for H3K9Me3 and −83 % for HP1γ in CNE-2 SHP-1 overexpression cells compared with CNE-2-empty vector cells (all *P* < 0.001) (Fig. 3c).

### Effects of SHP-1 knockdown in CNE-1 cells and overexpression in CNE-2 cells on NPC cell cycle distribution

**Fig. 4:**
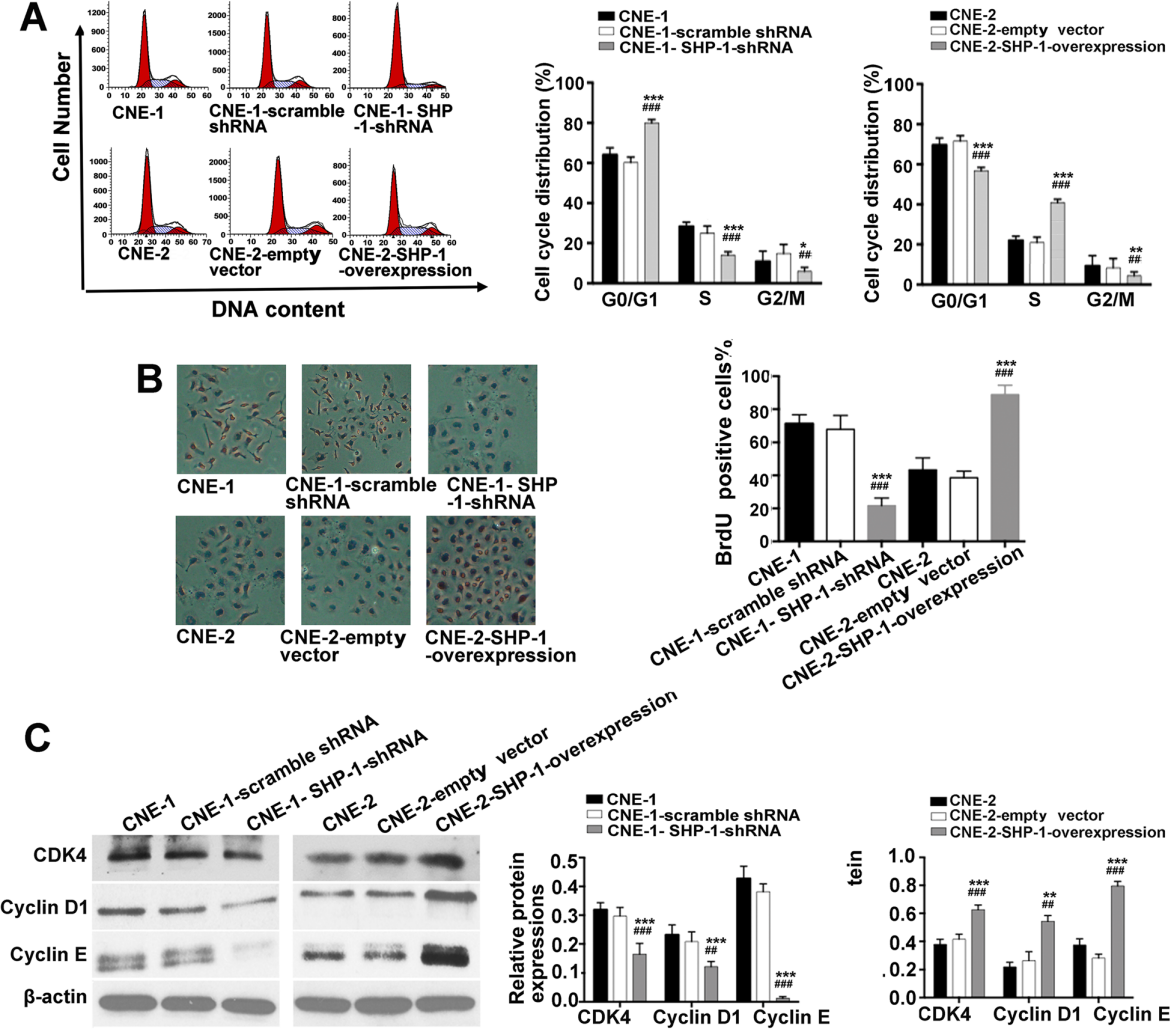
Effects of SHP-1 knockdown in CNE-1 cells and overexpression in CNE-2 cells on cell cycle distribution and cell cycle-related protein (CDK4, Cyclin D1 and Cyclin E) expressions. **a** Cell cycle was determined by flow cytometry using propidium iodide staining three days after transduction. **b** BrdU incorporation assay to monitor S phase progression (magnification: ×200). **c** Cell cycle-related protein expressions were determined by western blot. β-actin was used as an inner control. Data are shown as mean ± SD. *P < 0.05, ^**^ *P* < 0.01, ^***^ *P* < 0.001 vs. CNE-1 or CNE-2; ^##^ *P* < 0.01, ^###^ *P* < 0.001 vs. CNE-1- scramble shRNA or CNE-2-empty vector

As shown in Fig. 4a, compared with CNE-1-scramble shRNA cells, CNE-1 SHP-1 shRNA cells had a higher proportion of cells in G0/G1 (80.0 ± 1.7 % vs. 60.3 ± 2.7 %, *P* < 0.001), and lower proportions of cells in S (14.0 ± 1.7 % vs. 25.0 ± 3.6 %, *P* < 0.001) and G2/M (6.0 ± 2.0 % vs. 14.8 ± 4.5 %, *P* = 0.004) phases. Compared with CNE-2-empty vector cells, CNE-2 SHP-1 overexpression cells had lower proportions of cells in G1 (55.7 ± 2.6 % vs. 71.8 ± 2.9 %, *P* < 0.001) and G2/M (4.7 ± 0.8 % vs. 8.18 ± 1.3 %, *P* < 0.001) phases, and a higher proportion of cells in S phase (39.7 ± 2.2 % vs. 20.1 ± 2.9 %, *P* = 0.001).

The BrdU assay was used to monitor S-phase progression. Results showed that fewer cells were in the S phase in CNE-1 SHP-1 shRNA cells compared with CNE-1-scramble shRNA cells (21.6 ± 4.7 vs. 67.8 ± 8.4 cells, *P* < 0.001), while more cells were in the S phase in CNE-2 SHP-1 overexpression cells compared with CNE-2-empty vector cells (88.85 ± 5.6 vs. 38.6 ± 4.0 cells, *P* < 0.001) (Fig. 4b).

Compared with CNE-1-scramble shRNA cells, CNE-1 SHP-1 shRNA cells showed decreased expressions of CDK4 (−44 %, *P* < 0.001), cyclin D1 (−41 %, *P* = 0.001) and cyclin E (−97 %, *P* < 0.001). On the other hand, compared with CNE-2-empty vector cells, CNE-2 SHP-1 overexpression cells showed increased expression of CDK4 (+41 %, *P* < 0.001), cyclin D1 (+90 %, *P* = 0.001), and cyclin E (+124 %, *P* < 0.001) (Fig. 4c).

### Effects of SHP-1 knockdown in CNE-1 cells and overexpression in CNE-2 cells on p16/pRb pathway in NPC cells

**Fig. 5:**
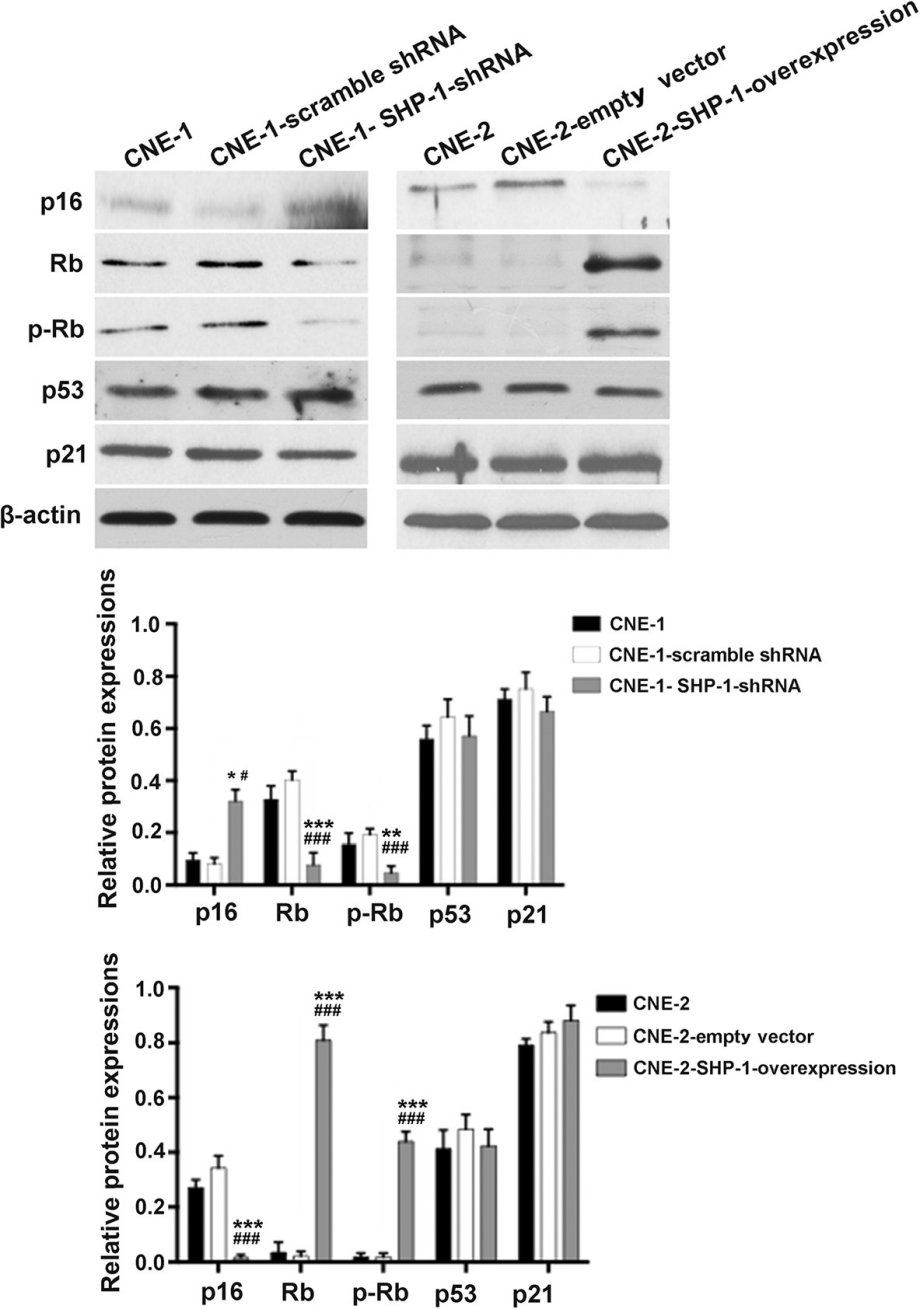
Effects of SHP-1 knockdown in CNE-1 cells and overexpression in CNE-2 cells on senescence and cell cycle-related signaling molecules (p16, Rb, p-Rb, p53, p21) expression. Protein expressions were determined by western blot. β-actin was used as control. Data are shown as mean ± SD. *P < 0.05, ^**^ *P* < 0.01, ^***^ *P* < 0.001 vs. CNE-1 or CNE-2; ^#^ *P* < 0.05, ^###^ *P* < 0.001 vs. CNE-1- scramble shRNA or CNE-2-empty vector

Compared with CNE-1-scramble shRNA cells, CNE-1 SHP-1 shRNA cells showed increased expression of p16 (+120 %, *P* = 0.02), and decreased expressions of Rb (−79 %, *P* < 0.001) and pRb (−76 %, *P* = 0.001). On the other hand, compared with CNE-2-empty vector cells, CNE-2 SHP-1 overexpression cells showed decreased expression of p16 (−95 %, *P* < 0.001), and increased expressions of Rb (+358 %, *P* < 0.001) and pRb (+248 %, *P* < 0.001) (Fig. 5). Levels of p53 and p21 were unchanged in both cell lines (all *P* > 0.05).

## Discussion

The aim of the present study was to assess the role of SHP-1 in the radioresistance and senescence of NPC cell lines. Results showed that SHP-1 downregulation resulted in increased senescence, increased radiosensitivity, higher proportion of cells in G0/G1, decreased expression of CDK4, cyclin D1, cyclin E, Rb, and pRb, and increased expression of p16. On the other hand, overexpression of SHP-1 resulted in decreased senescence, decreased radiosensitivity, higher proportion of cells in S-phase, increased expression of CDK4, cyclin D1, cyclin E, Rb, and pRb, and decreased expression of p16.

SHP-1 has recently emerged as a useful diagnostic marker and a potential target for therapeutic intervention in several malignancies because of its functional involvement in controlling cell proliferation and tumor cell cycle distribution [29]. Several studies have reported aberrant expression of SHP-1 in different cancers including NPC [16, 15, 23, 14], but no functional study has yet been reported in NPC. SHP-1 overexpression has been reported in NPC and associated with a worse prognosis [23]. Results of the present study showed that SHP-1 is involved in the regulation of the cell cycle and cellular senescence in NPC cells. In addition, SHP-1 levels were higher at baseline in the CNE-1 cells compared with the CNE-2 cells, and the CNE-1 cells showed higher radioresistance. This is supported by previous studies showing that the CNE-2 cell line has been shown to be less radioresistant than CNE-1 [27], and that the DNA repair mechanisms seem to be more efficient in the CNE-1 cell line [27, 28]. The results of the present study suggest that SHP-1 might play a role in radioresistance.

A previous study demonstrated that SHP-1 downregulates p16 expression, which in turn interacts and stabilizes FGFR1, thereby promoting epithelial-to-mesenchymal transition and therefore aggressive metastatic behavior of NPC cells [23]. Cellular senescence is an important mechanism for preventing the proliferation of potential cancer cells [18, 19]. Cellular senescence critically depends on two powerful tumor suppressor pathways: the p53 and pRb/p16INK4a pathways [22, 21, 20]. Both pathways integrate multiple aspects of cellular physiology to determine and orchestrate cell fate. Senescence growth arrest usually depends on the activation of the CDK inhibitors (p21CIP1 and p16INK4A) [30, 31], components of the tumor-suppressor pathways governed by p53 and pRb, and is generally associated with a G0/G1 cell cycle arrest. SAHF formation coincides with the recruitment of heterochromatin proteins and Rb to E2F-responsive promoters and is associated with the stable repression of E2F target genes [32]. Notably, both SAHF formation and the silencing of E2F target genes depend on the integrity of the Rb pathway and do not occur in reversibly arrested cells [33]. Often lost in a variety of malignancies, p16 acts as an allosteric inhibitor of the CDK4/6 complex to prevent its interaction with cyclin D1, inducing cell cycle arrest and senescence by activating the Rb pathway [34]. The CDK4/6-cyclin D1 complex-mediated phosphorylation and inactivation of Rb allows the transcription of E2F-dependent various cell cycle regulatory genes including cyclin E [34].

In the present study, stable suppression of SHP-1 mRNA in CNE-1 cells resulted in increased radiosensitivity compared with the parental cells, a decrease in the number of cells in S-phase and an increase in the expression of p16. Furthermore, we observed that SHP-1 increased cell proliferation by modulating cell cycle regulatory proteins like p16, CDK4 and cyclin D1. SHP-1 silencing suppressed growth via a unique mechanism involving G0/G1 cell cycle arrest. Interestingly, SHP-1 silencing in NPC cells resulted in cellular senescence, as suggested by cell morphology, increased SA-β-Gal-stained cells, and SAHF formation, which are considered to be characteristics of senescent cells [35]. Although the involvement of p16 in cellular senescence and its downregulation in NPC is well established [23], there is still a lack of comprehensive studies about its role in NPC senescence. Increased proliferation is mostly driven by altered cell cycle progression. In the present study, downregulation of SHP-1 resulted in decreased proliferation and G0/G1 cell cycle arrest, providing evidence that SHP-1 is a modulator of the cell cycle. Decreased proliferation and cell cycle arrest was associated with downregulation of cell cycle regulatory proteins such as CDK4 and cyclin D1. However, modulation of SHP-1 expression had no effect on expression levels of p53 and p21, two proteins that play important roles in cell cycle progression [36]. Cyclin E/D downregulation might be a direct consequence of SHP-1 depletion, but further study is necessary to elucidate this issue.

The present study is not without limitations. Indeed, we could not investigate the PTEN/p27KIP1 pathway in the present study. In addition, only cell lines were used, and the observation of SHP-1 in actual tumors should be beneficial. However, we plan to study this association in the near future.

## Conclusions

In conclusion, we observed that SHP-1 downregulation or overexpression affected radioresistance, cell senescence and cell cycle distribution in NPC cell lines. These findings not only offer new perspectives in the modulation of senescence by SHP-1, but also provide strong evidence for SHP-1-based therapeutic interventions in NPC patients.

## Abbreviations

ANOVA: Analysis of variance
HP1γ: Heterochromatin protein-1γ
NPC: Nasopharyngeal carcinoma
RT: Radiotherapy
SD: Standard deviation
SNK: Student-Newman-Keuls
